# Bariatric surgery as a treatment for refractory obesity in patients with schizophrenia. Weight-loss outcomes and safety in 36 months follow-up

**DOI:** 10.1192/j.eurpsy.2022.644

**Published:** 2022-09-01

**Authors:** P. Lorencetti, E. Chaim, E. Cazzo, A. Junior, P. Dalgalarrondo

**Affiliations:** 1 State University of Campinas, Psychiatry Department, Campinas, Brazil; 2 State University of Campinas, Surgery Department, Campinas, Brazil

**Keywords:** bariatric surgery, obesity, schizophrénia

## Abstract

**Introduction:**

Obesity has increased worldwide and concerns comorbidity in patients with schizophrenia, and is linked to a high mortality rate in this group. Although bariatric surgery is the gold standard treatment for refractory obesity, it rarely is indicated for subjects with schizophrenia due to psychotic symptoms recurrence.

**Objectives:**

Report weight-loss outcome and psychopathology changes over 36 months follow-up of 5 patients with schizophrenia submitted to bariatric surgery.

**Methods:**

Patients have been followed for 36 months. Clinical and anthropometric assessments such as percentage of excess weight loss (EWL) and body mass index (BMI) have been performed at 6, 12, 24, and 36 months follow-up. The Positive and Negative Syndrome Scale (PANSS) was used to assess psychopathology status. Wilcoxon test was used to assess statistical differences.

**Results:**

The sample included four female and one male subject, with BMI at baseline 42,81± 5,66. The results of BMI and EWL over time are described in Table 1. A significant statistical difference was found between BMI at baseline and at T6, T12, T24, and T36 (p<0,05). EWL was higher at T12 ( compared with T6), but not different from other measurements. PANSS scores at the baseline were 7,7 ± 1,6 for a positive domain, 8,7 ± 2,3 for a negative domain, and 19,2 ± 6 for general psychopathology, with no statistically significant differences during the follow-up.

**Figure:**
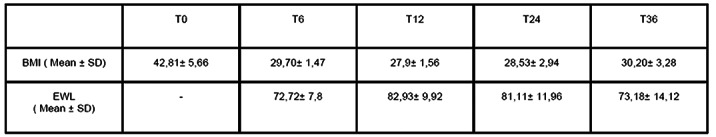

**Conclusions:**

Despite the small sample, bariatric surgery has been shown a safe and efficient refractory obesity treatment in patients with schizophrenia.

**Disclosure:**

No significant relationships.

